# P011: Clinical utility of initial follow-up blood cultures in patients with catheter-related Staphylococcus aureus bacteremia

**DOI:** 10.1186/2047-2994-2-S1-P11

**Published:** 2013-06-20

**Authors:** MS Lee, KH Park, MH Jung, YS Kim

**Affiliations:** 1 Internal Medicine, Kyung Hee University Hospital, Kyung Hee University School of Medicine, Seoul, Korea, Republic Of; 2 Infectious Diseases, Asan Medical Center, University of Ulsan College of Medicine, Seoul, Korea, Republic Of

## Introduction

Limited data was available on clinical relevance of routine blood culture follow-up in patients with catheter-related *S. aureus* bacteremia (CRSAB). The aim of this study was to determine the clinical relevance of performing follow-up blood culture (BC) follow-up in patients with CRSAB.

## Methods

All patients with CRSAB were prospectively included between August 2008 and December 2010. During the study period, infectious disease specialists strongly encourage the follow-up BCs performed between 48 and 96 hours after onset of bacteremia. Complication was considered related to SAB if they were recorded during the antibiotic treatment for the SAB and confirmed by radiology and/or culture of *S. aureus* from a normally sterile site. Recurrence was defined as the isolation of *S. aureus* from the bloodstream or other sterile body site during the 12-week post-treatment follow-up period.

## Results

Of the 217 patients with CRSAB, follow-up BCs were performed in 175 patients (81%) between 48 and 96 hours. Of these 175 patients, follow-up BCs were positive in 74 (42%) and negative in 101 (58%) patients. Follow-up BCs was more like to have positive results in episodes of CRSABs caused by methicillin-resistant isolates than those caused by methicillin-susceptible isolates (86.5% vs. 57.3%; *P* < 0.001). Cardiac echocardiography to detect infective endocarditis was more likely to be performed in patients with positive follow-up BCs than in patients with negative follow-up BCs (87.8% vs. 68.3%; *P* = 0.003). Complication occurred in 54% of patients with positive follow-up BC results and in 13% of patients with negative follow-up BC results (*P* < 0.001). Eight (18%) of the 74 patients with positive follow-up BC result experienced the recurrence, but 1 (1%) of the 111 patients with negative follow-up BC result experienced recurrence (*P* = .005).

## Conclusion

In patients with CRSAB, initial follow-up BCs were clinical predictors for complication and recurrence. This practice is simple and useful tool to guide the extent of diagnostic evaluation and duration of treatment in these patients.

## Disclosure of interest

None declared

